# Unified Deep Learning of Molecular and Protein Language Representations with T5ProtChem

**DOI:** 10.1021/acs.jcim.5c00051

**Published:** 2025-04-08

**Authors:** Thomas Kelly, Song Xia, Jieyu Lu, Yingkai Zhang

**Affiliations:** † Department of Chemistry, New York University, New York, New York 10003, United States; ‡ Simons Center for Computational Physical Chemistry at New York University, New York, New York 10003, United States; § NYU-ECNU Center for Computational Chemistry at NYU Shanghai, Shanghai 200062, China

## Abstract

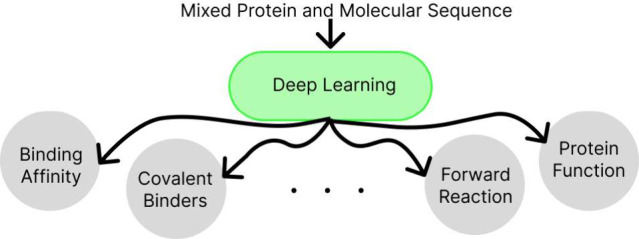

Deep learning has revolutionized difficult tasks in chemistry and biology, yet existing language models often treat these domains separately, relying on concatenated architectures and independently pretrained weights. These approaches fail to fully exploit the shared atomic foundations of molecular and protein sequences. Here, we introduce T5ProtChem, a unified model based on the T5 architecture, designed to simultaneously process molecular and protein sequences. Using a new pretraining objective, ProtiSMILES, T5ProtChem bridges the molecular and protein domains, enabling efficient, generalizable protein-chemical modeling. The model achieves a state-of-the-art performance in tasks such as binding affinity prediction and reaction prediction, while having a strong performance in protein function prediction. Additionally, it supports novel applications, including covalent binder classification and sequence-level adduct prediction. These results demonstrate the versatility of unified language models for drug discovery, protein engineering, and other interdisciplinary efforts in computational biology and chemistry.

## Introduction

Deep learning models have made significant breakthroughs in modeling and generative abilities in chemistry and biology.^1−8^ Models such as AlphaFold-3,^9^ ESM-Fold,^10,11^ and RoseTTAFold All-Atom have improved protein folding and a protein complex prediction.^12^ Many models are closely derived from the transformer and other similar language models.^13−17^ For instance, ESM-2 and -3^10,11^ or ProtGPT2^18^ are aimed at proteins tasks. Similarly in chemistry, models like ChemBERTa,^19,20^ T5Chem,^21^ SELFormer,^22^ and BARTReact have been developed to predict chemical properties, reactions, and other problems.^23^ Moreover, the convenience of natural language for users has led to the development of models trained on both the natural language and the scientific domains.^3,24−27^

Generally, protein language models are trained on protein sequences, which are represented as a string of single letter amino acid codes. In chemistry, meanwhile, language models are often trained on molecular sequences, typically in the Simplified Molecular Input Line Entry System (SMILES) format,^28^ but other chemical languages can be used such as Self-Referencing Embedded Strings (SELFIES),^29−31^ InChI,^32^ or DeepSMILES.^33^

Despite the difference in languages for expressing molecular and protein sequences, both share an underlying atomic representation, motivating the search for a unified language model. Relying solely upon canonical amino acids limits sequence expressiveness, as proteins exist in a variety of complexes and can contain a variety of modifications. Nonetheless, representing the protein as a sequence of SMILES, or any chemical language, is inefficient, since the average protein sequence is between 300 to 400 amino acids long.^34^ If a language model can learn the shared atomic representation between proteins and chemical sequences, it can provide a more efficient and generalizable model.

Beyond efficiency, a growing body of research shows that various pretraining objectives can enhance performance on downstream tasks.^35,36^ Pretraining models is a computationally expensive process, but it is essential to achieve state-of-the-art performance on downstream tasks. Much of the pretraining focus has been in a single domain or combining and extending two or more domains, such as text, protein, and molecules.^21,37^ Many models implement RoBERTa pretraining styles,^38^ where the model is trained on a masked language model objective.^10,11,19,21^

Multidomain models have also been developed, like MolT5, BioT5+, and Galactica, which cover the largest breadth of domains, including natural language, mathematics, code, amino acid sequences, DNA sequences, and chemical sequences.^3,24−26^ Galactica, MolT5, and BioT5+ follow pretraining techniques closely related to T5, where the model is trained on masked spans.^24,26,39^ Very few models specifically target modeling both chemistry and proteins. Flam-Shepherd et al. developed a generative model that learns from the atomic level of proteins using SELFIES, while Pei et al. developed two multimodal models that train on natural language, molecular, and protein sequences, but they did not explore training on the underlying atomic structure.^39−41^ In drug design, models that capture both representations of proteins and chemicals are of great interest. Other work, such as Electra-DTA and DeepDTA, use separate pretrained models for chemistry and proteins,^42,43^ followed by further architecture to combine the two during the fine-tuning process.

**Figure 1:**
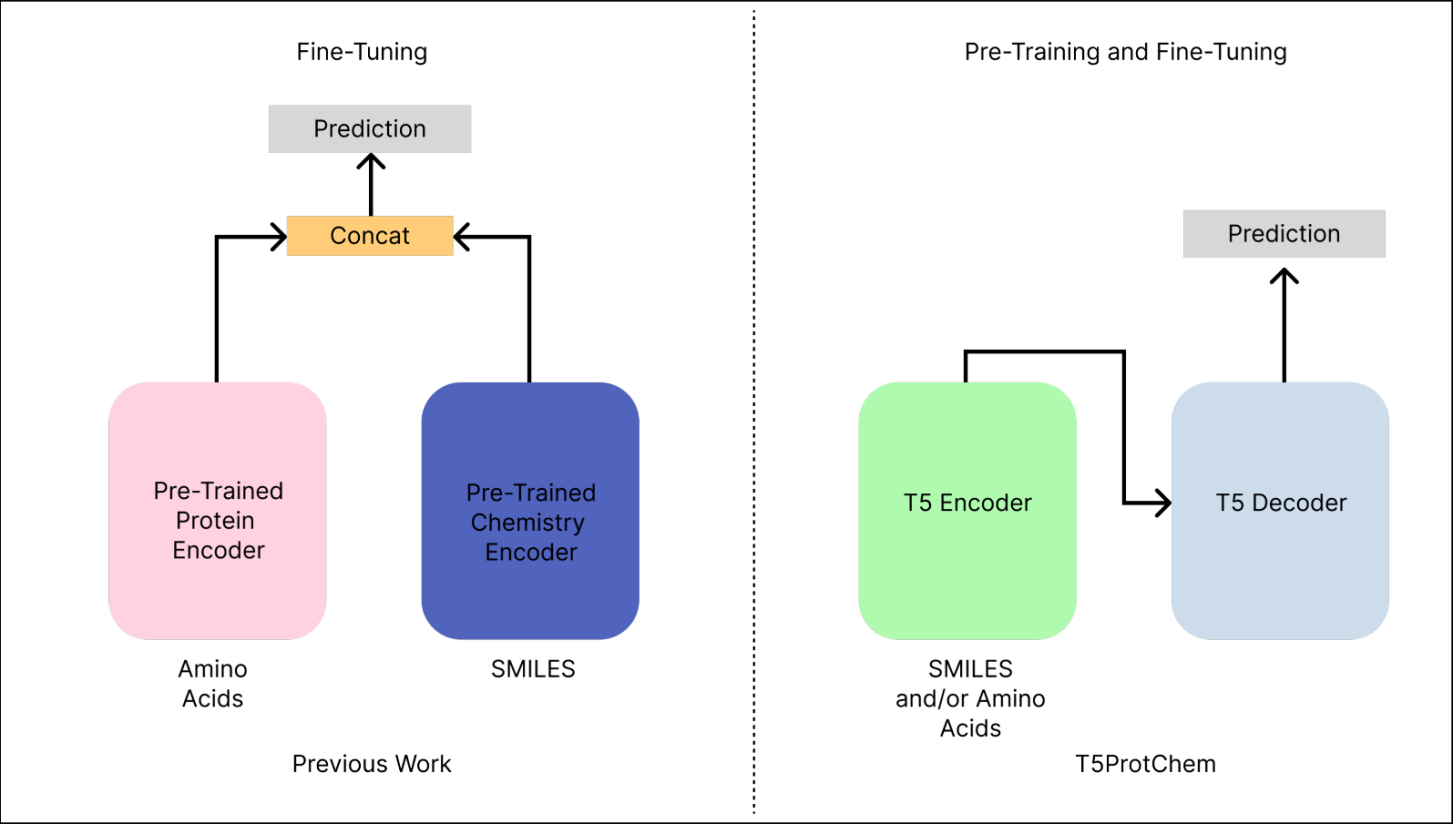
Many deep-learning models have distinct chemistry and protein blocks. Our model learns a shared representation by pretraining on unmasking the corresponding SMILES sequences of masked SMILES and amino acid sequences. This improves the generalizability and ease of use of the model. This also enables fine-tuning for protein, molecular, and joint protein-molecular tasks.

**Figure 2:**
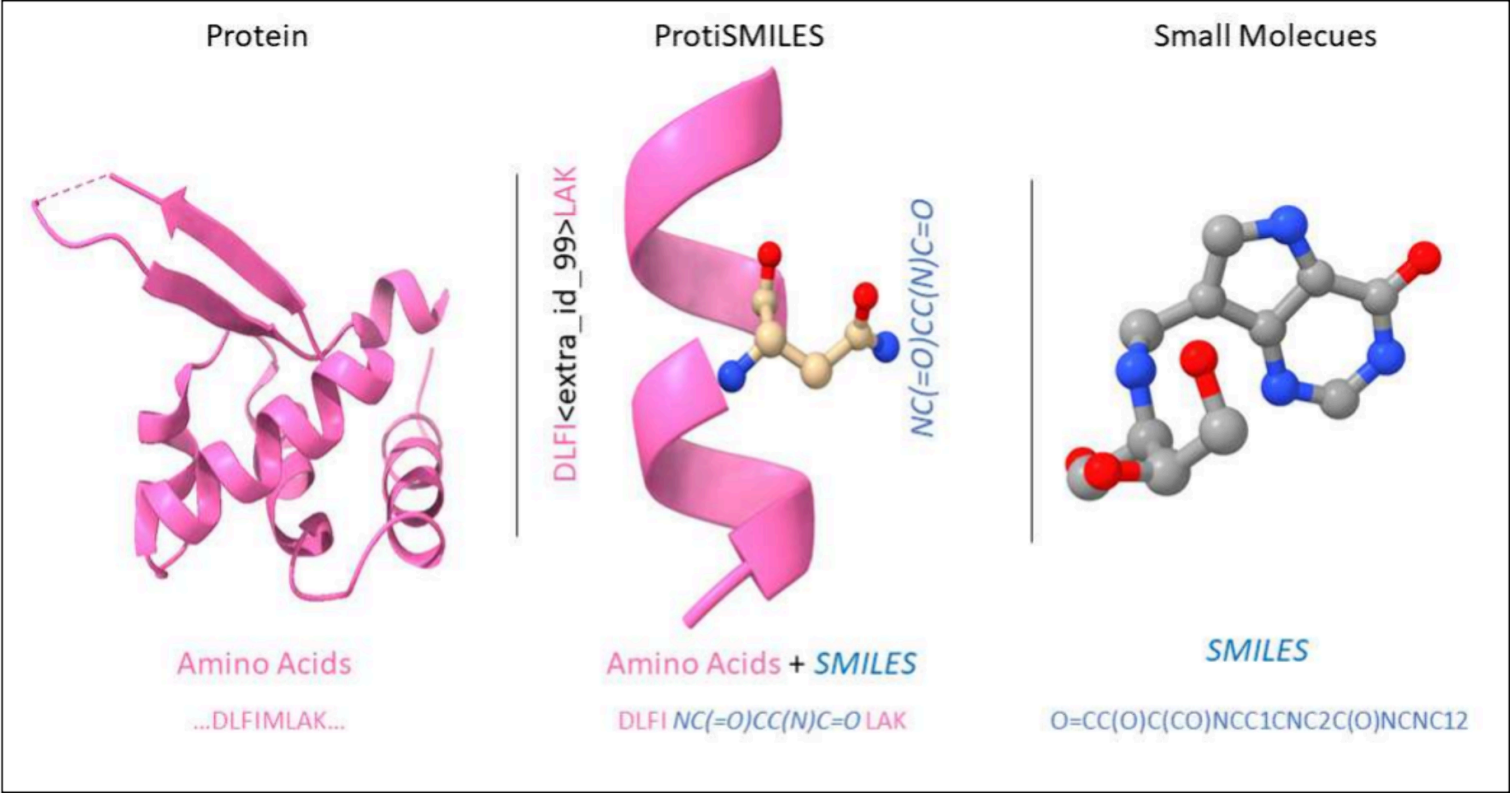
Proteins and chemicals sequences are expressed as single letter amino acid codes and SMILES, but share a common atomic representation. We illustrate the shared atomic structure ProtiSMILES seeks to exploit. In practice, portions of the amino acid sequences are masked. Subsequently, the decoder is tasked with decoding the masked amino acid sequence with the corresponding SMILES sequence. The model can still be trained on small molecules, using their SMILES sequence.

In this work, we introduce T5ProtChem, a new pretrained model designed to process protein and chemical sequences simultaneously (Figure 1). Building on the foundational methods of T5 and T5Chem,^21,35^ we incorporate a new ProtiSMILES objective into the self-supervised framework (Figure 2). This adaptation enables the encoder to process span-masked amino acid sequences while the decoder generates the corresponding SMILES sequences, effectively bridging the “translation” between these languages. We train T5ProtChem by leveraging data from both PubChem and UniRef50 to construct a diverse pretraining data set comprising hundreds of millions of samples with molecules of varying lengths, producing a single pretrained model capable of protein, molecular, and protein-molecular tasks.^44,45^ To demonstrate the versatility of our unified model, we evaluate it across a range of tasks, including protein function prediction, forward reaction prediction, and binding affinity estimation. Additionally, we introduce and test a new sequence-based covalent binder task, where we predict the result adduct’s SMILES sequence and position using a newly curated data set.

## Methods and Data Sets

**Figure 3:**
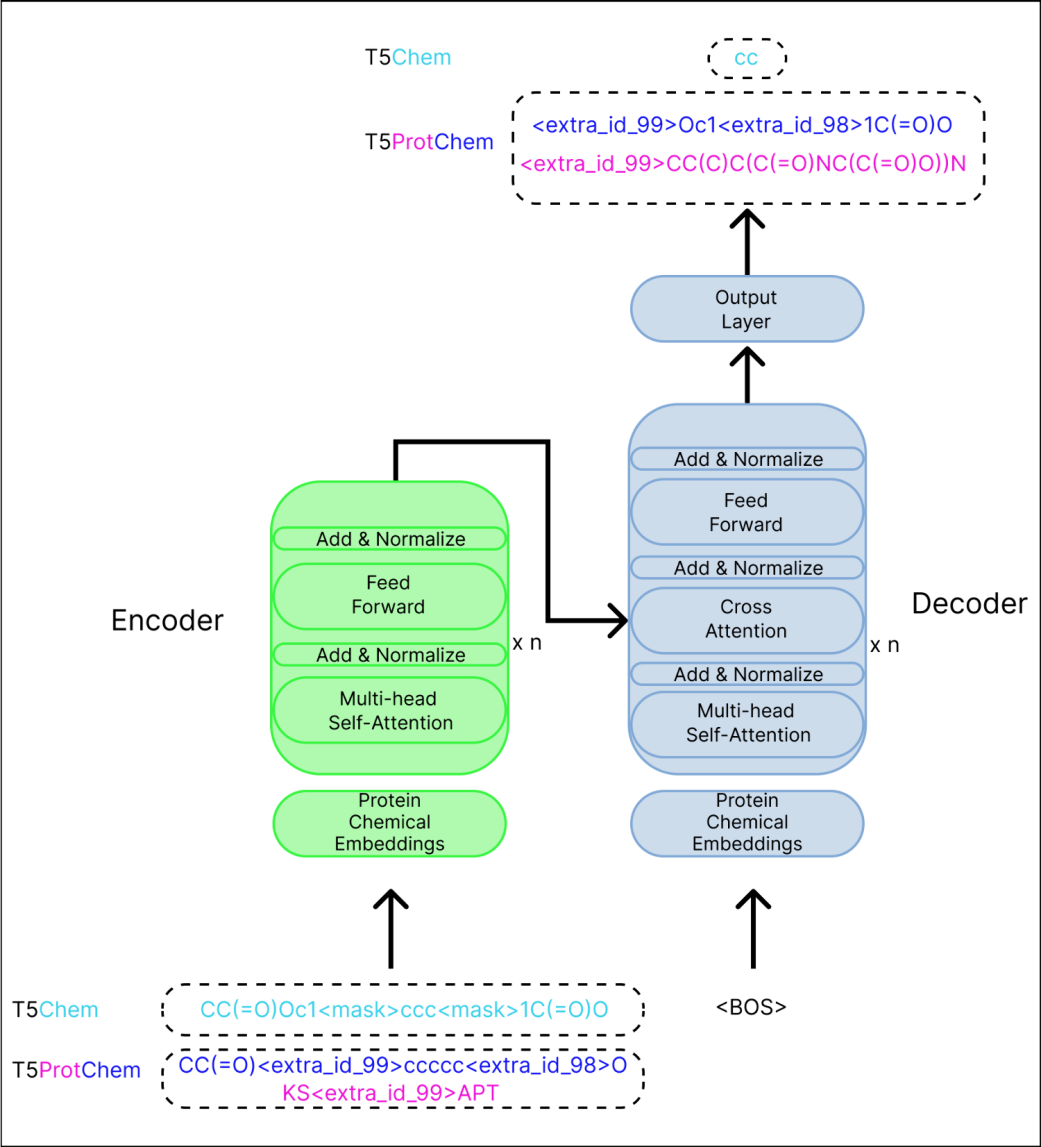
Differences between T5Chem and T5ProtChem are indicated by the dashed boxes, along with T5ProtChem’s expanded protein and chemical vocabulary. While T5Chem and T5ProtChem can both take SMILES sequences as input, T5ProtChem can handle protein sequences shown in pink. Furthermore, T5ProtChem mixes both protein and chemical sequences during pretraining combined with the new ProtiSMILES pretraining objective, highlight by the pink amino acid sequence being translated to its corresponding SMILES sequence, also in pink. Moreover, T5Chem utilizes single token masks, T5ProtChem uses span-masking with sentenial tokens, denoted as decreasing <extra_id_##> tokens.

T5ProtChem builds off the advancements made in T5Chem,^21^ but with several changes. While T5ProtChem is still a T5 model, like T5Chem, T5ProtChem subscribes more closely to pretraining techniques introduced in T5, including sentinel tokens and span-masking.^35^ In contrast, T5Chem only uses token level masking.^21^ Furthermore, our model learns in both the protein and chemical domains, allowing inputs of either amino acids or SMILES strings. During pretraining, we use a new objective, ProtiSMILES, to connect the two languages, by decoding masked amino acid sequences to SMILES sequences (Figure 3). This allows us to complete protein, chemical, and protein-chemical tasks with a single pretrained model.

### Tokenization and Pretraining

Unsupervised masking objectives are fundamental to pretraining language models and other deep learning architectures, as they enable models to learn robust representations and capture semantic relationships without explicit supervision. This approach is beneficial for leveraging unlabeled data during pretraining, which enables more performant fine-tuning on tasks with limited labeled data.^35,46^

In our model, T5ProtChem, we adopt an encoder-decoder architecture inspired by the Text-to-Text Transfer Transformer (T5)^35^ (Figure 3). Our model employs character-level tokenization across both protein and chemical sequences. To address the challenge of distinguishing amino acid tokens from similar symbols in SMILES, we prepend canonical amino acids with a <P> prior to tokenization, and treat the resulting amino acid, <P>S for example, as a single token. This explicit differentiation allows the model to seamlessly separate the two domains without confusion, streamlining the learning process.

During pretraining, we use a span-masking objective on SMILES sequences, replacing spans of tokens with sentinel tokens arranged in descending order (e.g., <extra_id_99>). Approximately 15% of the tokens are masked, with an average span length of three. The corrupted sequences are processed by the bidirectional encoder, and the decoder reconstructs the masked spans. To facilitate this, we introduce the Span-Mask: tag at the beginning of each sequence.

**Figure 4:**
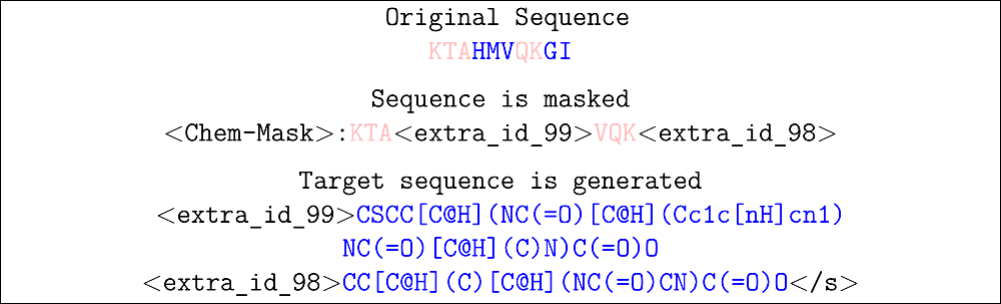
An example of the ProtiSMILES objective. The amino acids are masked by extra id tokens, and then result is unmasked as the SMILES sequence of the amino acids.

Expanding on this methodology, we develop the ProtiSMILES objective, which extends span-masking to amino acid sequences. Here, we extract a contiguous segment of up to 128 amino acid tokens, apply the same 15% masking probability, and then task the decoder with generating the corresponding SMILES sequences instead of amino acids. To indicate this mode, we prepend a Chem-Mask: token rather than a Span-Mask: token. We illustrate this process using an example protein sequence (Figure 4).

The model’s vocabulary consists of 203 tokens, encompassing basic special tokens, such as <pad>, <unk>, and <eos>, as well as specialized sentinel tokens for pretraining. By combining character-level tokenization, domain-specific modifications, and masking objectives, T5ProtChem effectively bridges the gap between SMILES and protein sequences, enabling it to learn meaningful representations across both domains.

We curate an expansive pretraining data set to leverage this capability to process both protein and chemical sequences. For the ProtiSMILES objective, we used the UniRef50 data set,^45^ equivalent to ESM-2 for pretraining.^10^ The training set contained 52156140 protein sequences while we withheld 1661660 proteins, identical to ESM-2. We used a subset of 27119 protein sequences of the 500000 samples from the AlphaFold DB Swiss-Prot data set (AF-500 K) for ProtiSMILES validation, which contains sequences spanning 48 organisms.^47^ We used PubChem for our chemical pretraining data set, which contains the 97479896 SMILES sequences,^44^ the same data set in T5Chem (Table 1).^21^ Similarly, we kept out 50000 molecules for validation.

**Table 1:** Pretraining Datasets Used for T5ProtChem^a^

data type	training size	validation size	training source	validation source
chemicals	97479896	50000	PubChem	PubChem
protein	52156140	27119	UniRef-50	AF-500 K

a The pretraining relies only on SMILES sequences and protein sequences. The decoder is only tasked with producing the SMILES sequence, not amino acids sequences. Model cross entropy performance measured on protein datasets.

Following the architecture laid out in T5, the model has eights layers, ten attention heads, and an embedding size of 640. The feed-forward layers have dimensions of 2048. In total, the model has 102 M learnable parameters. We train the model for 142599 steps using bf16 precision on 4 Nvidia A100 80 GB GPUs with an effective batch size of 2880 and learning rate of 1.414e-4 with cross entropy loss. With these parameters, our best model took 8 days to train.

### Fine-Tuning

An effective pretrained model can be fine-tuned on a variety of tasks. With our unified pretrained model, we first investigate forward reaction prediction and protein function prediction, common chemical and protein specific tasks. We utilize the same data set as T5Chem for the forward reaction prediction task,^21^ a sequence to sequence task. The model is given the SMILES sequence of the reactants and reagents, and generates the resulting product SMILES sequence. For forward reaction prediction, we fine-tune the model for 29 epochs with a batch size of 128 and a learning rate of 0.0005 with a constant learning rate schedule. molecular function data set from DeepFRI for protein function prediction, where there are 489 Genetic Ontology (GO) terms to describe a protein’s function (Table 3). Here, the model receives a protein sequence and predicts the 489 class multilabel GO term vector. With protein function prediction, the model is fine-tuned for 237 epochs with a batch size of 24 and a learning rate of 0.00005 with cross entropy loss (Table 4).

**Table 2:** Description of the Covalent Binder Dataset

data set	adduct and position task	covalent binder classification task
train	3401	6802
validation	425	850
test	425	850

**Table 3:** Description of Fine-Tuning Datasets

task	binding affinity	forward reaction	protein GO term
source	electra-DTA bindingDB^42^	USPTO_MIT^50^	DeepFRI^51^
train	115620	409035	29902
validation	14452	40000	3323
test	14453	30000	3416

**Table 4:** Fine-Tuning Hyperparameters for Various Tasks

task	epochs	batch size	learning rate	loss type
protein function prediction	237	24	0.00005	cross entropy
forward reaction prediction	29	128	0.0005	cross entropy
binding affinity prediction	42	50	0.00005	KL divergence
covalent binder classification	39	40	0.00005	binary cross entropy
covalent binder position and adduct prediction	110	20	0.00005	cross entropy

We evaluate whether the model can utilize the connection between chemical and protein languages by testing protein–drug binding affinity prediction. We use the refined BindingDB data set from Electra-DTA, a model that also implements a novel strategy and architecture for jointly training on protein and molecular samples.^42^ Our architecture is designed to process protein–drug interactions by having the encoder generate the ligand representation SMILES sequences, while the decoder processes the protein amino acid sequences, T5ProtChem autoregressively generates a new representation that is then passed to a regression head.

The BindingDB data set, from ELECTRA-DTA,^42^ contains 115620 protein–drug pairs in the training set. We select the best performing model based on the root mean squared error (RMSE) of the validation set, which contains 14452 protein–drug pairs. We test on an additional 14453 protein–drug pairs. For binding affinity prediction, we fine-tune the model for 42 epochs with a batch size of 50 and a learning rate of 0.00005 with KL Divergence loss (Table 4).

Covalent binders are an advantageous drug modality because the drug often has a longer duration of action. The drug forms a nonreversible covalent bond with an amino acid in its target protein. The resulting adduct is an non-natural amino acid, which limits usage of current language models that solely rely on natural amino acids.^10^ By leveraging the CovBinderInPDB data set, we curated a data set of 6783 covalent binders and the corresponding protein sequence.^48^ We highlight the versatility of T5ProtChem by classifying covalent and noncovalent binders and also by predicting the adduct SMILES and binding position on this data set.

We utilize GroupSELFIES to connect the corresponding amino acid with the adduct group to generate the data set.^30^ By replacing the dummy atom in the adduct SMILES in the CovBinderInPDB database with the appropriate valency, we are able to connect arbitrary amino acids with arbitrary adducts. However, GroupSELFIES does not provide this feature intuitively for all adducts, so we are limited to describing singly-bonded adducts. Additionally, we exclude adducts that bond to two amino acids in the sequence. In total, we exclude 592 pairs, or 8%, of the CovBinderInPDB data set.

Several entries are proteins complexes with identical binders on identical chains. Therefore, we split the data set only on the 4251 unique protein-binder pairs. We then randomly split these by 80% for training, 10% for validation, and 10% for testing. In the training data set, there are 3401 protein–drug pairs. There are 425 pairs in both the validation data set and test data set, respectively. For the binary covalent binder classification, we mix an equal number of noncovalent pairs, serving as the negative samples, with the covalent pairs, sourced from the BindingDB data set (Table 2).^42^ We also use the CovalentInDB data set of 2,665 covalent pairs not seen in training as an external test set for testing our classifier.^49^

For training model on the covalent binder classification task, we fine-tuned on the sequence classification data set for 39 epochs with a constant learning rate of 0.00005 with a batch size of 40 using binary cross entropy loss. The model is shown a protein and ligand sequence and then predicts whether it is a covalent binder or not, with binary classification. Training the model to predict both the position and adduct required 110 epochs with a batch size of 20 and learning rate of 0.00005, while using cross entropy loss (Table 4). We designed the task as a sequence-to-sequence task, where model is shown the protein and SMILES sequence, and then generates the resulting protein sequence with the covalent binding adduct SMILES, being the amino acid bonded with the ligand expressed as the chemically valid SMILES, bounded by the <extra_id_99> and <extra_id_98> tokens respectively. This also allows us to benchmark the accuracy of the residue position of the protein.

## Metrics

Our model’s performance on forward reaction prediction is based on the accuracy on the product sequence generated.

We measure performance of protein function prediction using the commonly used *F*_max_ metric for the Molecular Function domain after we train and test on the data set from DeepFRI.^51^
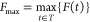
1where
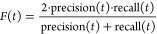
2and
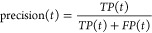
3
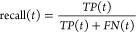
4

Here:

- *T* is the set of all possible threshold values (logit scores).
- *TP*(*t*) is the number of true positives at threshold *t*.
- *FP*(*t*) is the number of false positives at threshold *t*.
- *FN*(*t*) is the number of false negatives at threshold *t*.

Moreover, we also evaluate the area under the precision–recall curve.
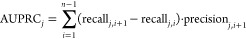

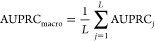
We evaluate the performance of our model on the binding affinity prediction task using metrics proposed in several other studies. We evaluate the ability of our model to correctly order the binding affinity of protein–drug pairs using the Concordance Index (CI) eq 5.^43,52^
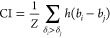
5Let *Z* represent the number of protein–drug pairs *B* arranged in such a way that the true binding affinity of each pair is greater than that of the subsequent pair. *h*(*x*) is a step function.
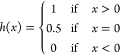
6

We also evaluate our models using the root mean squared error (RMSE) metric, Pearson correlation coefficient, the coefficient of determination, and eq 7. *r*_*m*_^2^ combines the traditional *r*^2^ metric with a penalization for the discrepancy between the predicted and true values, incorporating both model fit and predictive power.
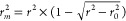
7where *r*^2^ is the traditional coefficient of determination and *r*_0_^2^ is defined according to Roy et al.^43,53^

For the covalent binder classification task, we report accuracy, F1 score, precision, and recall. For the adduct position and sequence prediction task, we report the accuracy of the sequence and position.
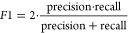
8
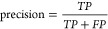
9
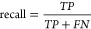
10

## Results and Discussion

Our unified model demonstrates versatility by performing well across tasks traditionally tackled by separate domain specific models. By leveraging the ProtiSMILES objective to bridge between protein and molecular representations, our model is pretrained to handle both amino acid and SMILES sequences addressing challenges at the intersection of chemistry and biology.

We first evaluate performance of the model on forward reaction prediction, a frequently benchmarked task of chemical reasoning. Here, the model predicts the reaction product given the reactants and reagents of a single step reaction. Despite this being a domain typically served by specialized chemical models, our unified model achieves state-of-the-art performance (Table 5), surpassing established baselines such as the Molecular Transformer and T5Chem.^21,54^ This result demonstrates the benefits of integrating protein and molecular pretraining, even for tasks traditionally confined to chemical domains.

**Table 5:** Forward Reaction Prediction

model	accuracy % (Top-1) *↑*	accuracy % (Top-2) *↑*	accuracy % (Top-5) *↑*
molecular transformer^54^	88.8	92.4	94.2
T5Chem	89.5	92.9	95.2
T5ProtChem	89.7	93.7	95.7

Next, we assess the model’s capacity for protein function prediction by focusing on Gene Ontology (GO) terms within the Molecular Function category. Unlike methods that do integrate structural information (e.g., DeepFRI^51^), our model relies solely on sequence data. While domain specific models like ESM-2 exhibit higher *F*_max_ scores, our approach still achieves competitive performance (Table 6). Moreover, external ESM-2 embeddings can be fed to the T5ProtChem decoder, achieving superior results than ESM-2. More importantly, by handling proteins and molecules within the same framework, our model can readily extend its capabilities to new tasks.

**Table 6:** Protein Function Prediction for Molecular Function^a^

model	*F*_max_ *↑*	AUPRC
DeepFRI^51^	0.540	0.434
ESM-2	0.621	0.573
DeepGO^51,55^	0.485	0.310
T5ProtChem	0.549	0.444
T5ProtChem and ESM-2	0.626	0.573

a We use 150M parameter ESM-2 model for comparison.

We demonstrate the versatility of our model on protein-drug binding affinity prediction, a critical domain for drug discovery. Prior approaches often rely on separate pretrained models specialized for proteins or small molecules.^42,43^ In contrast, our model benefits from a unified representation that captures the underlying molecular and amino acid patterns, enabling it to outperform these specialized baselines on the BindingDB data set (Table 7). The ability to excel in this biochemical task reinforces the practical value of a single model trained across domains.

**Table 7:** Model Achieves State-of-the-Art Performance on the Refined BindingDB Dataset from Wang et al.^42^^,^^a^

model	RMSE *↓*	CI *↑*	R *↑*	*r*_*m*_^2^ *↑*
DeepDTA^42^	0.912	0.812	0.824	0.623
DeepCDA^42^	0.919	0.822	0.808	0.631
Electra-DTA^42^	0.832	0.832	0.852	0.645
T5Chem + ESM-2	0.923	0.814	0.828	0.580
T5ProtChem	0.809	0.840	0.862	0.650

a We combine a pretrained T5Chem with 150M parameter ESM-2 model.^10,21^.

**Figure 5:**
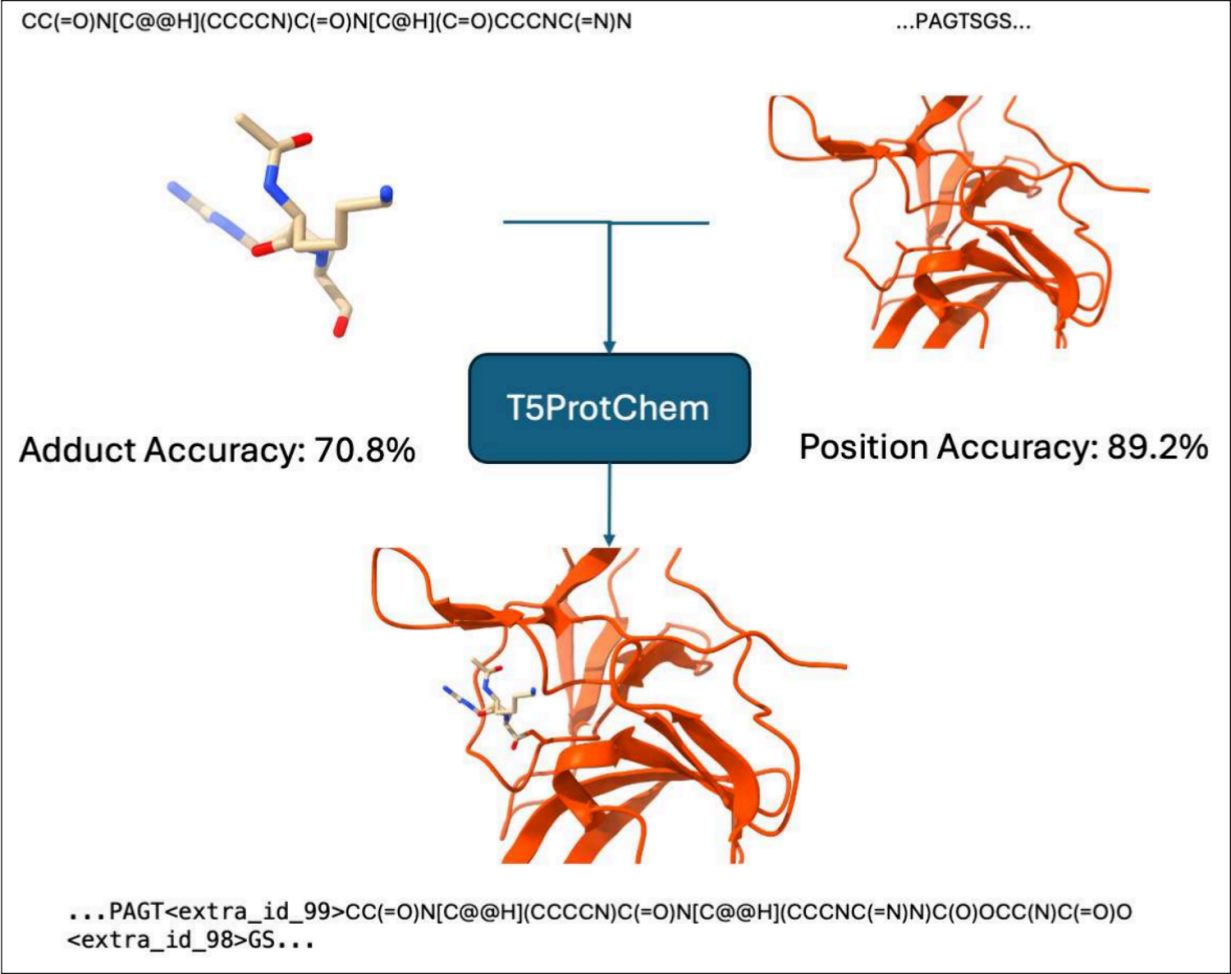
Example of the covalent binder prediction. The model can generate both the correct adduct with 70.8% and the position of adduct within the sequence at 89.2% accuracy.

To further test the ability of our model, we use our novel sequence-based covalent binder data set derived and expanded from CovBinderInPDB to predict the resulting SMILES adduct sequence and residue number of the covalent binding site. These covalent binders have garnered substantial interest due to their enhanced selectivity and potency.^48,56−60^ Our model demonstrates strong performance in these tasks, achieving 70.8% accuracy for predicting the covalent adduct and 89.2% accuracy for identifying the correct residue position (Table 2, Figure 5). This level of performance on this new task that requires an integrated understanding of both protein and chemical sequences further illustrates the advantage of unifying these domains under a single pretraining regime.

Finally, by incorporating an equivalent number of noncovalent pairs from the BindingDB data set,as the negative samples, we expanded the training and evaluation sets to further test the robustness of the model The resulting performance, combined with high accuracy on an external set of covalent pairs from CovalentInDB, confirms that our approach generalizes beyond single data sets (Table 8).^49^

**Table 8:** Covalent Binder Classification

data set	accuracy	recall	precision	F1 score
CovBinderinPDB^48^	99.6	1.0	0.993	0.996
CovalentInDB^49^	84.9	0.850	1.00	0.919

## Conclusion

We present T5ProtChem, a unified language model pretrained on both chemical and protein sequences, demonstrating strong performance in forward reaction prediction, protein function prediction, and binding affinity prediction. We also introduce a new benchmark using a sequence-based covalent adduct prediction task, highlighting the model’s ability to generalize across domains traditionally requiring separate models.

By leveraging a shared atomic representation between proteins and chemicals, our new ProtiSMILES approach integrates these domains, providing a foundation for fine-tuning on diverse downstream applications. This work establishes T5ProtChem as a versatile tool for tasks at the intersection of protein and chemical modeling, offering a pathway for more integrated approaches in drug discovery research.

## Data Availability

The code to reproduce these experiments is available at https://github.com/tkella47/T5ProtChem.git. All data, pretraining and fine-tuning, along with model weights are available at 10.5281/zenodo.13984057

## Supporting Information Available

Table S1: Overview of tasks with type, input, and output. Table S2: Pretraining ablation results comparing models trained from scratch versus pretrained models. Figures S1–S3: Attention heatmaps for protein and molecule interactions. Figure S1: Average self-attention heatmap for protein function prediction. Figures S2 and S3: Cross-attention heatmaps for binding affinity prediction, with Figure S3 showing an alternate visualization (PDF)
