# Supplementary material for: Unified Deep Learning of Molecular and Protein Language Representations with T5ProtChem
Source: J Chem Inf Model. 2025 Apr 8;65(8):3990–8. doi: 10.1021/acs.jcim.5c00051 (PMC12042257; doi:10.1021/acs.jcim.5c00051)
Supplement: Supplementary file 1 — ci5c00051_si_001.pdf [file ci5c00051_si_001.pdf]

Supporting Information: Unified Deep Learning of Molecular and Protein Language Representations with T5ProtChem

Thomas Kelly,† Song Xia,† Jieyu Lu,† and Yingkai Zhang*,†,‡,¶

†*Department of Chemistry, New York University, New York, NY 10003, United States*

‡*Simons Center for Computational Physical Chemistry at New York University, New York, NY 10003, United States*

¶*NYU-ECNU Center for Computational Chemistry at NYU Shanghai, Shanghai 200062, China*

E-mail: yingkai.zhang@nyu.edu

Table S1: Overview of tasks with type, input, and output.

Task	Type	Input	Output
Forward Reaction	Seq2Seq	Reactants and Reagents (SMILES)	Products (SMILES)
Protein Function Classification	Multilabel Classification	Protein Sequence	Multi-label GO Term Vector
Binding Affinity	Regression	Protein Sequence and SMILES Sequence	Binding Affinity
Covalent Binder Classification	Binary Classification	Protein and SMILES Sequence	Binary Output
Covalent Adduct and Position Prediction	Seq2Seq	Protein and SMILES Sequence	Protein and SMILES Sequence

Table S2: Pre-training ablation.

Model	Accuracy % (Top-1) ↑	Accuracy % (Top-2) ↑	Accuracy % (Top-5) ↑
From Scratch	0.00	0.00	0.00
With Pre-training	**89.7**	**93.7**	**95.7**

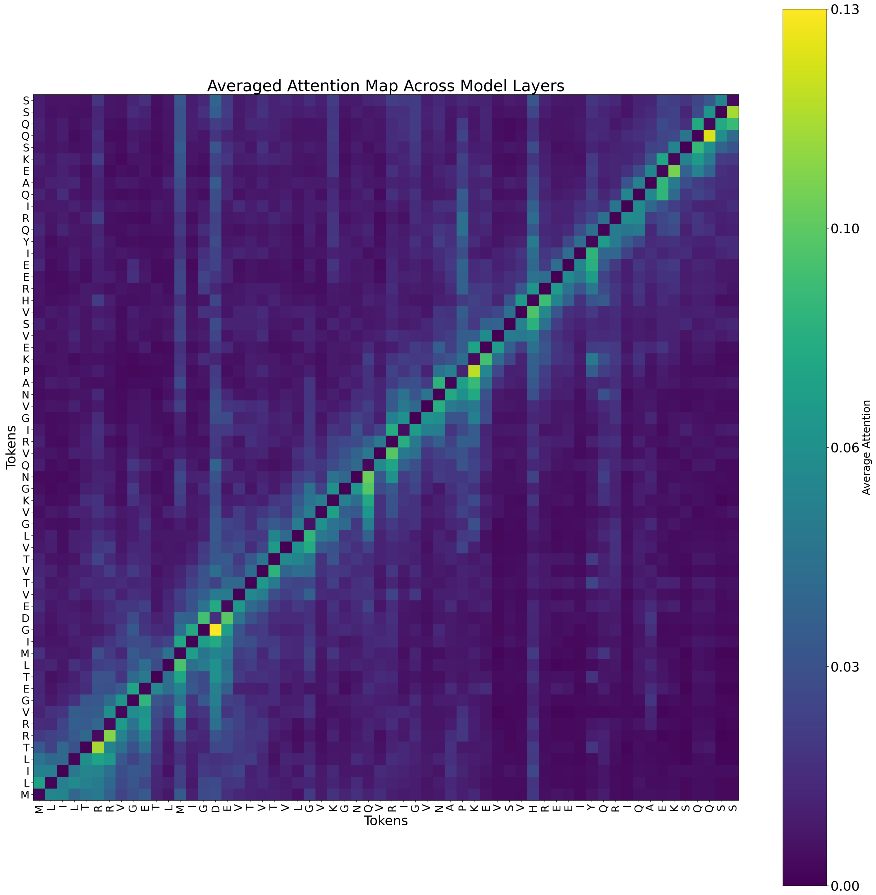

Figure S1: Average self-attention heatmap for protein function prediction.

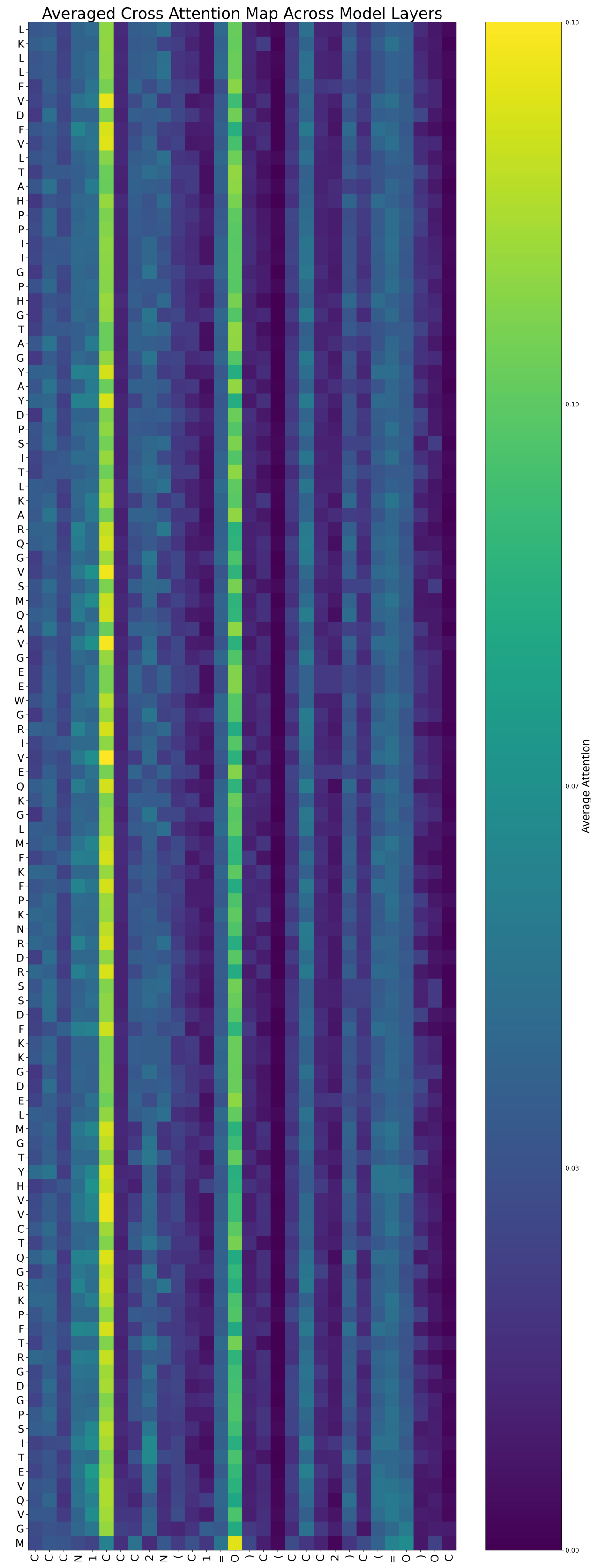

Figure S2: Cross-attention heatmap for binding affinity prediction.

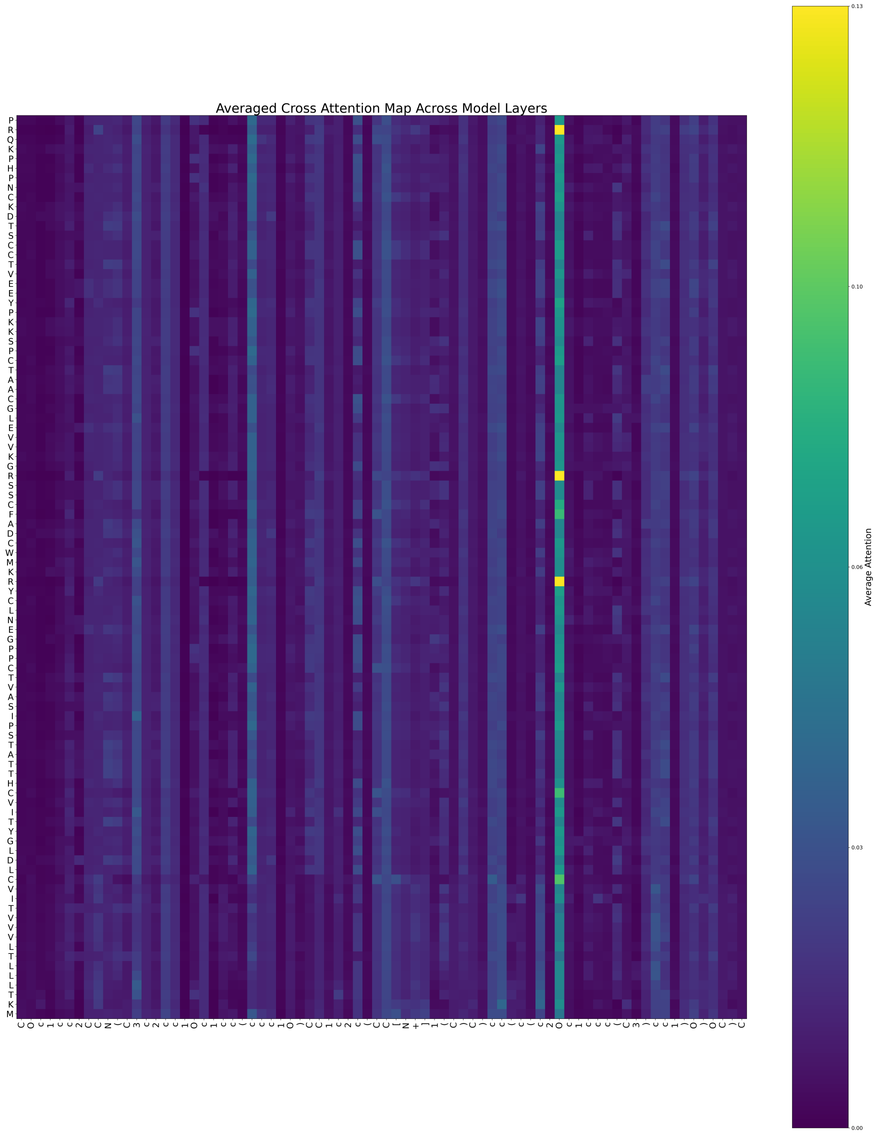

Figure S3: Alternate visualization of cross-attention for binding affinity prediction.
